# Prognosis of incompletely resected small rectal neuroendocrine tumor using endoscope without additional treatment

**DOI:** 10.1186/s12876-022-02365-z

**Published:** 2022-06-09

**Authors:** Boram Cha, Jongbeom Shin, Weon Jin Ko, Kye Sook Kwon, Hyungkil Kim

**Affiliations:** Department of Internal Medicine, Digestive Disease Center, Inha University School of Medicine, Inha University Hospital, 27 Inhang-ro, Jung-gu, Incheon, 400-711 Republic of Korea

**Keywords:** Rectal neuroendocrine tumor, Incomplete endoscopic resection, Local recurrence

## Abstract

**Background:**

In recent years, the incidence of rectal neuroendocrine tumors (NET)s has markedly increased due to the widespread use of screening colonoscopy. However, many patients are referred from local clinics after undergoing conventional endoscopic mucosal resection (EMR) for polyps without perceived NET, with a pathological report of incomplete resection. We evaluated the prognosis of incompletely resected small rectal NET without additional endoscopic resection for small rectal NET less than 10 mm in diameter present within the submucosal layer showing good prognosis, due to its rare metastatic potential.

**Methods:**

We retrospectively reviewed patients from 2008 to 2018 at a single center who had had small rectal NET (located in the rectum from the anal verge to 20 cm in proximity) and had undergone ‘incomplete resection’ using endoscopy with a positive deep margin or with a very small safe deep margin (< 100 um). A small rectal NET was defined as a tumor ≤ 10 mm in diameter, without lymph node nor distant metastasis, and with low grade (G1) according to the WHO grading system.

**Results:**

Of 267 patients who were diagnosed with small rectal NET, 77 were diagnosed with incomplete resection or possible remnant NET. Of those, 55 patients (55/77, 71.4%) were referred from local clinics post EMR diagnosed as polyps. The rate of histologically incomplete resection was highest in endoscopic submucosal dissection (11/21, 52.4%) and lowest in surgical resection (0/9, 0%), while endoscopic submucosal resection with band ligation showed an incomplete resection rate of 4.4% (5/113). After exclusion of 36 patients, namely 21 patients had undergone additional surgical (n = 6) or endoscopic (n = 15) resection and 25 patients who were lost during the follow-up period of 2 years, 31 patients had undergone surveillance with endoscopic evaluation or either a biopsy or radiological evaluation for distant metastasis during a median follow-up duration of 2 years. None of the incompletely resected small rectal NET patients showed local or distant metastasis.

**Conclusion:**

Incomplete resection of small rectal NET with G1 grade has a good prognosis without additional treatment.

**Supplementary Information:**

The online version contains supplementary material available at 10.1186/s12876-022-02365-z.

## Background

Recently, the incidence of rectal neuroendocrine tumors (NET)s has increased worldwide due to the increase in the rate of screening colonoscopy [1, 2]. Most rectal NETs are localized at the time of diagnosis and have a low metastatic potential [3]. Rectal NETs are mostly small, with 66% being less than 10 mm in diameter [2]. Due to the rare incidence of rectal NET (almost 1 case per 100,000 adults) [4], many patients who had undergone conventional endoscopic mucosal resection (EMR) [5] were referred from the local clinic with the diagnosis of polyps without perceived NET. Thus, they have an incompletely resected NET in their pathological reports.

During the assessment of its therapeutic efficacy, incomplete resection of NET is of major concern to clinicians due to its possible poor prognosis. Macroscopically, complete resection is achieved in most cases [6, 7]; however, a microscopically remnant NET may still be present on resection margins. A remnant NET can either result in local recurrence or distant metastasis; accordingly, additional resection or endoscopic examination may be required. However, factors influencing incomplete resection were inversely correlated with small tumor size (< 10 mm), invasion within submucosa, absence of lymphatic invasion [8–10], and low grade tumor (G1) -according to the WHO grading system [11]. Furthermore, there is a very low metastatic risk associated with a tumor size smaller than 10 mm [2, 12].

European guidelines recommended annual follow-up in perpetuity in case of incomplete resection of G1 grade tumors less than 10 mm in diameter, and within mucosa or submucosa [13]. A newly updated NCCN guideline recommended a 6- to 12-month follow-up for incompletely resected G1 grade tumors ≤ 10 mm in diameter. After that period, if there is no evidence of a residual disease, no further follow-up is recommended [14]. Guidelines on surveillance and salvage therapy for residual NET after incomplete resection are limited. Consequently, clinicians follow different strategies due to the lack of consensus on a consolidated therapeutic approach for dealing with incompletely resected NETs.

Since small rectal NETs ≤ 10 mm in diameter show good prognosis due to their rare metastatic potential, the aim of this study was to evaluate the prognosis of incompletely resected small rectal NETs that did not undergo additional endoscopic resection.

## Patients and methods

### Study design and study population

**Fig. 1:**
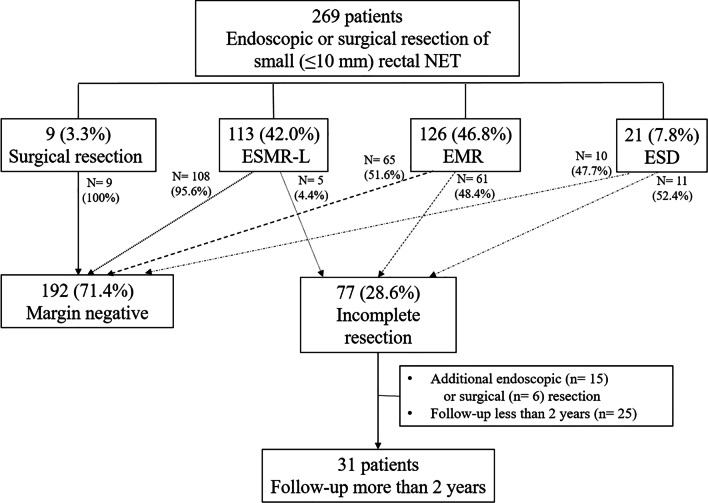
Algorithm of endoscopic resection for NET patients

The clinical data of patients pathologically diagnosed with small rectal NET who had undergone endoscopic resection between January 2008 and December 2018 at Inha University Hospital was retrospectively reviewed. Moreover, clinical data of patients who had undergone endoscopic resection in local clinics were also included if they were referred for incomplete resection of small rectal NET. Small rectal NET was defined as tumor ≤ 10 mm in diameter, without lymph node nor distant metastasis as confirmed by abdominal computed tomography (CT). The inclusion criteria were as follows: (1) age > 18 years at the time of treatment; (2) the result of incomplete resection include a positive deep margin or a very small safe margin (< 100 um); (3) at least 2 years of follow-up post-endoscopic resection. Twenty-one patients with additional endoscopic (n = 15) or surgical (n = 6) treatment and 25 patients with less than 2 years’ follow-up were excluded from this study. At least 2 years of observation with colonoscopy or CT was conducted in 31 patients (Fig. 1).

### Endoscopic procedures

**Fig. 2:**
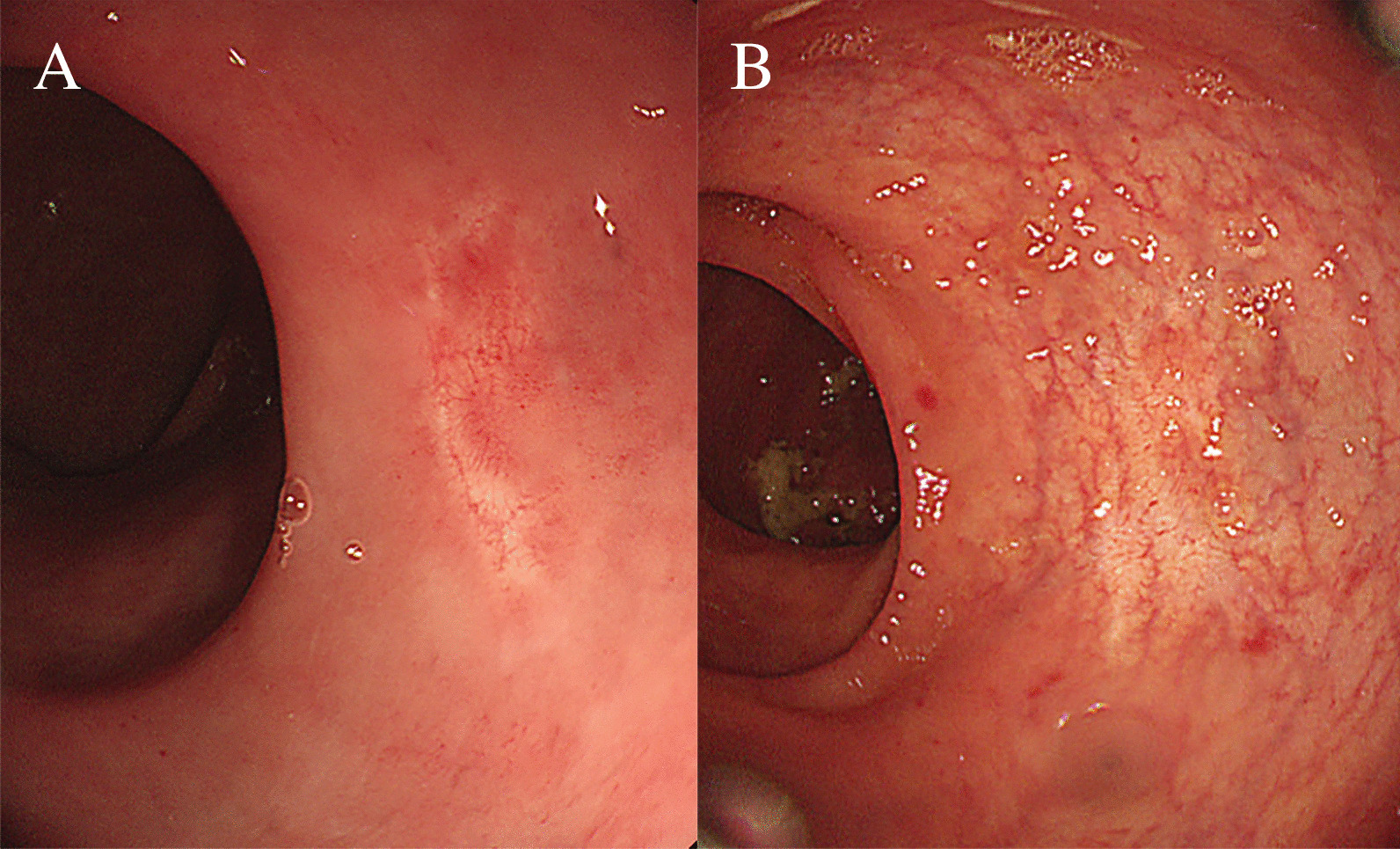
**A** Case of post polypectomy scar from local clinic, **B** Follow-up after 2 years

**Fig. 3:**
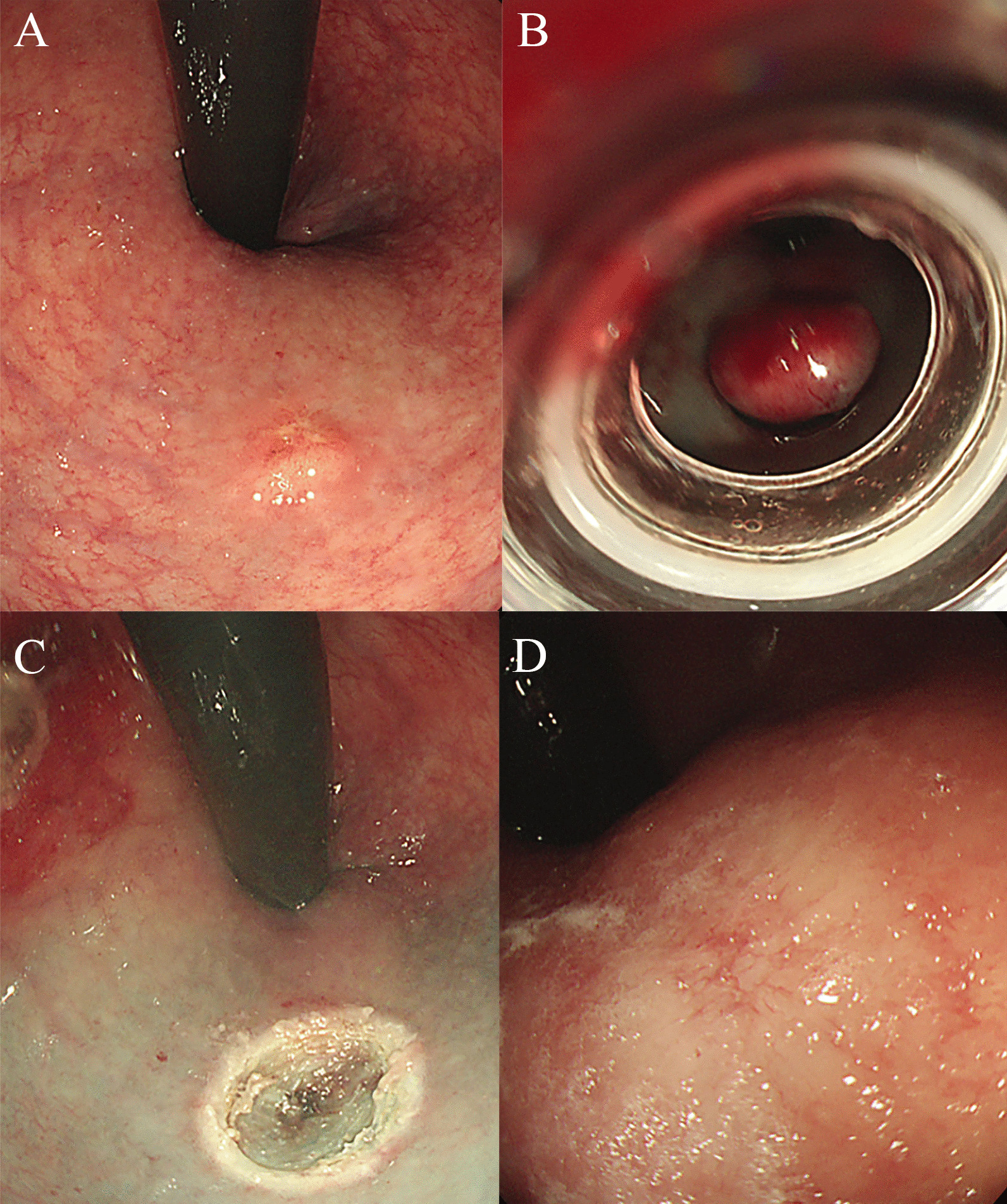
**A** Biopsy-confirmed rectal NET, **B** ESMR-L, **C** Post-resection, **D** Follow-up after 2 years

**Fig. 4:**
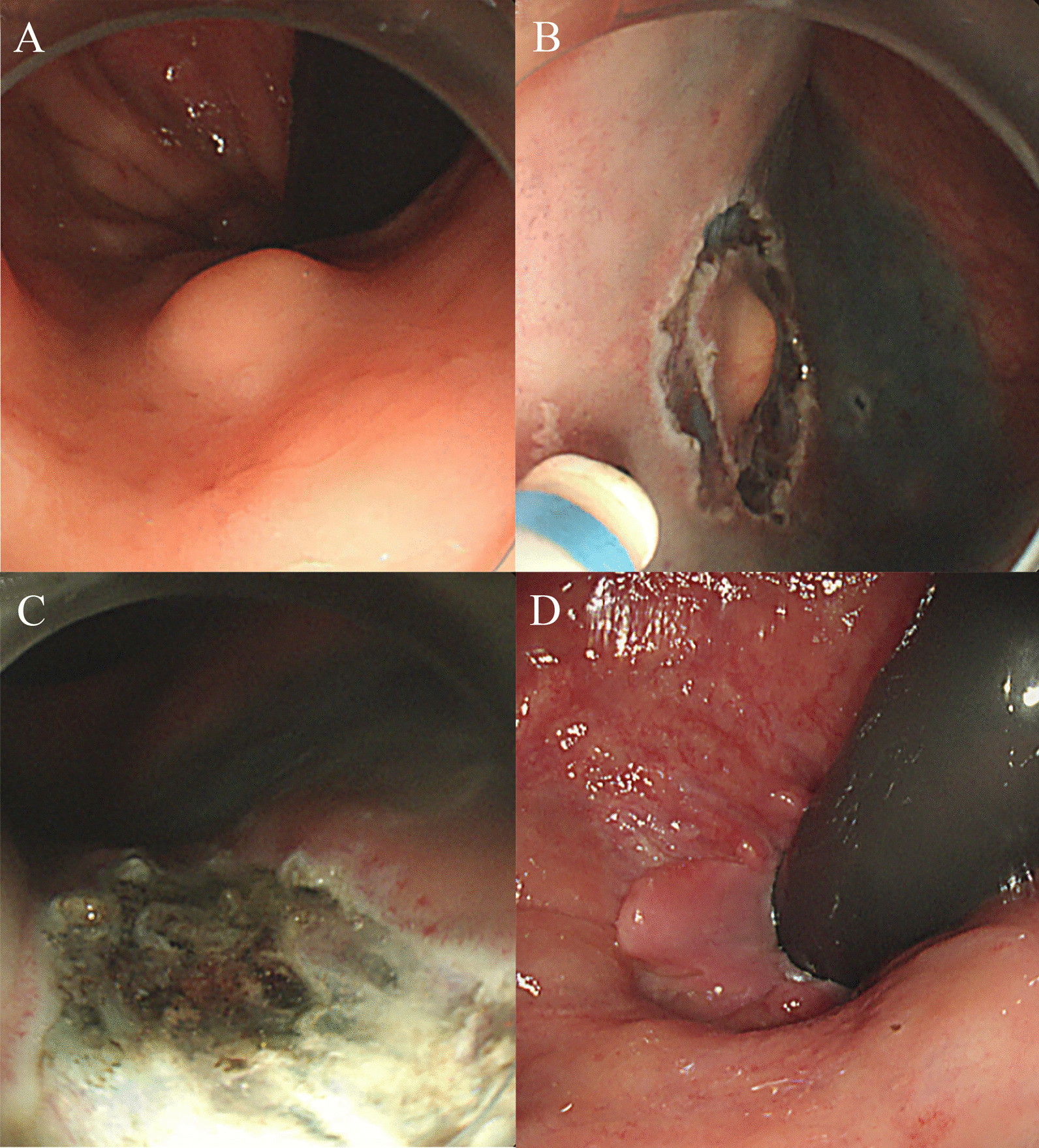
**A** Initial endoscopy, **B** ESD, **C** Post-resection, **D** Follow-up after 7 years

Endoscopic ultrasonography (EUS; GF-UC240P-AL5, Olympus Optical Co., Tokyo, Japan) was performed at Inha University Hospital prior to endoscopic resection to determine the depth of NET with respect to the muscular layer. The optimal endoscopic resection techniques for rectal NETs were decided and performed by endoscopists having more than 5-years’ experience in endoscopic submucosal dissection (ESD) Fig. 2. The resection methods were classified as follows: conventional EMR, modified EMR including cap-assisted EMR or precut-EMR, endoscopic submucosal resection-ligation (ESMR-L) (Fig. 3A, B, C and D), and ESD (Fig. 4A, B, C and D). The ESMR-L technique was performed as previously reported by our department [15]. A polypectomy snare (Olympus Optical Co., Tokyo, Japan) was used for conventional or modified EMR and ESMR-L. For ESD and precut EMR, Dual Knife (KD-650Q, Olympus Optical Co., Tokyo, Japan) was used for circumferential incision and submucosal dissection.

### Histopathological examination

Resected specimens were histologically evaluated using light microscopy to determine histological type, depth of invasion, lateral and vertical resection margin involvement, and lymphovascular invasion. Immunohistochemical staining for neuron-specific enolase and synaptophysin was performed to confirm the diagnosis. Mitotic count and Ki-67 index were assessed for determination of tumor grade according to the grading system of the WHO [11]. En bloc resection was defined as resection of the entire lesion in a single piece. Pathologically complete resection was defined as the absence of tumor cells’ involvement on the lateral and vertical margins of the resected tumor on microscopy. Resection margin was classified as positive, negative, or indeterminate, which included a very small safe margin where tumor cells were involved within a region of < 100 um from the tumor.

### Follow-up of patients

Patients having either histologically indeterminate or positive deep margin underwent follow-up colonoscopy to determine if a visible residual NET was present. Whenever a residual NET was suspected, an additional biopsy or EUS was performed. However, if the scar seemed clear (Fig. 2A), only observation by colonoscopy was performed during the follow-up period (Fig. 2B). In addition to colonoscopy, CT was performed to inspect distant metastasis.

### Statistical analysis

The clinical characteristics of the study subjects were expressed as medians (ranges) for continuous variables and numbers (percentages) for categorical variables. The differences between categorical or continuous variables were analyzed using Mann–Whitney U test, Student’s t-test, chi-square test, or Fisher’s exact test. The overall re-bleeding and cumulative survival rates were estimated using Kaplan–Meier. Two-tailed *p*-values < 0.05 were considered statistically significant. Statistical analyses were performed using SPSS v25.0 (SPSS Inc., Chicago, IL, USA).

## Results

### Comparison of complete and incomplete histological resection rates among different endoscopic procedures

In the period between January 2008 and December 2018, the rate of microscopic incomplete resection was 28.6%. This represents 77 patients from a total of 269 endoscopically or surgically resected small rectal NET patients. About 4.4% of ESMR-L (5/113), 48.4% of conventional EMR or modified EMR (61/126), and 52.4% of ESD (11/21) patients had undergone incomplete resection. This shows that the highest rate of complete resection is evident in ESMR-L (Fig. 1). Of the 77 patients with incomplete resection, 57 had undergone resection by conventional EMR (74.0%), 11 patients by ESD (14.3%), 5 patients by ESMR-L (6.5%), and 4 patients by modified EMR (5.2%). Among conventional EMR patients, about 75.4% of the lesions (43/57) were not initially suspected as NETs and thus were resected as polyps (Table 1).

**Table 1:** Endoscopic procedures in margin-positive small rectal NET

	Margin-positive (n = 77)
Conventional EMR	57(74.0%)
local refer, resection as polyp	43/57(75.4%)
resection as NET	14/57(24.6%)
Modified EMR (EMR-C/P)	4(5.2%)
ESMR-L	5(6.5%)
ESD	11(14.3%)

*N* number, *EMR* endoscopic mucosal resection, *NET* neuroendocrine tumor, *EMR-C/P* endoscopic mucosal resection-cap-assisted/precut, *ESMR-L* endoscopic submucosal resection-ligand, *ESD* endoscopic submucosal dissection

### Long-term prognosis

After a median follow-up period of 39.8 months (range 24.2–119.7), none of the patients with incomplete resection of small rectal NETs experienced local nor distant metastasis by CT scans. There was no mortality case in both groups (Table 2).

**Table 2:** Long-term outcome of local recurrence (n = 31)

Local recurrence	0 (0%)
Distant metastasis	0 (0%)
Initial follow-up period (in months)	12.5 (range, 0.4–52.0)
Last follow-up period (in months)	39.8 (range, 24.2–119.7)
Overall mortality	0 (0%)

*N* number

### Clinical and pathologic characteristics in incompletely resected small rectal NET without recurrence

Of the 31 patients, 19 were male (48%) and the median age was 54 years (range, 27–84). Nine had undergone conventional EMR (61.3%), while ESMR-L was used for 3 patients (9.7%), modified EMR was used for 5 patients (16.1%), and ESD was used for 4 patients (12.9%) (Table 3).

**Table 3:** Basic characteristics of margin-positive small rectal NET without recurrence (n = 31)

Characteristic	Value
Age (in years)	54 (range, 27–84)
Gender, male	19 (48%)
*Modes of endoscopic resection*	
Conventional EMR	19 (61.3%)
Modified EMR (EMR-C/P)	5 (16.1%)
ESMR-L	3 (9.7%)
ESD	4 (12.9%)

*N* number, *EMR* endoscopic mucosal resection, *EMR-C/P* endoscopic mucosal resection-cap-assisted/precut, *ESMR-L* endoscopic submucosal resection-ligand, *ESD* endoscopic submucosal dissection

The average size of small rectal NET was 5.2 ± 1.7 mm. Upon examining tissue pathology, all the tumors were found to be confined to the submucosa, except one which was partly found in a muscular propria. All of cases were of low-risk G1 grade as per the WHO’s classification. Among the 31 cases of incomplete resection, 5 patients (16.1%) had a very small safe margin and 4 patients (12.9%) were confirmed to have lymphovascular invasion after endoscopic resection (Table 4).

**Table 4:** Clinical characteristics of margin-positive small rectal NET without recurrence

	Value
Size (in mm)	5.2 ± 1.7
Depth	
within SM	30 (97.4%)
within MP	1 (3.2%)
Mitotic count/10 HPF	
< 2	31 (100%)
Ki-67 index (< 3%)	
< 1%	25 (80.6%)
≤ 1– < 2%	5 (16.1%)
≥ 2%	1 (3.2%)
Margin positive	
Yes	26 (83.9%)
No, but < 100 um	5 (16.1%)
Lymphovascular invasion (yes,%)	4 (12.9%)

*NET* neuroendocrine tumor, *SM* submucosal, *MP* muscular propria, *HPF* high power field

## Discussion

Our study demonstrated that incompletely resected small G1 grade rectal NET (≤ 10 mm) was safely followed up for at least 2 years without the need for further resection. Furthermore, ESMR-L showed the highest success rate in the complete resection of small rectal NET among all the other endoscopic resection methods.

Clinical characteristics of 21 excluded patients because of undergoing additional treatment were listed on Additional file 1: Table S1. Two patients of surgical resection (2/6) were determined high risk of rectal NET with grade 2, Ki 67 index of 3%. Two patients of additional endoscopic resection (2/15) were detected remnant tumor through EUS and 4 patients (4/15) were suspected remnant through endoscopic view. However, more than half of patients were undergone additional resection only with deep margin positive results even though clean scar was shown on endoscopy.

A special focus was directed at the incidental detection of rectal NET during screening colonoscopy at a local clinic. Among all the previously reported prognostic factors contributing to incomplete resection, the consistent factors were tumor diameter larger than 10 mm, depth in the muscular propria, and the presence of lymphovascular invasion [8–10]. Since EUS is not available in most local clinics, the decision should depend on the tumor size and the knowledge that the tumor is not a simple polyp but rather a subepithelial tumor which could be present in areas deeper than the mucosa.

Upon comparing different endoscopic procedures in terms of the rate of complete resection, ESMR-L procedure showed the highest rate (108/113, 95.6%) followed by ESD procedure (10/21, 47.7%) (Fig. 1). Our group has previously reported that ESMR-L had a significantly higher complete resection rate than ESD (53/53, 100% vs. 13/24, 54.2%, *p* < 0.001) in the resection of small rectal NET with significantly shorter procedure time (5.3 ± 2.8 min vs. 17.9 ± 9.1 min, *p* = 0.000) [15]. In other previous reports, vertical resection involvement was higher (6.5% to 19.4%) than lateral involvement (nearly 100%) [16–18] because NET is located on submucosa layer requiring higher skill. However, incomplete resection rate by ESD of 47.7% from our group is markedly higher than that of other groups. Lack of ESD experience of rectal NET might be one reason. Recently, Park et al. reported that there was not a significant difference between the resection rates of ESD and transanal endoscopic microsurgery in rectal NET with size < 10 mm in diameter, (83.5% vs. 93.9%, *p* = 0.063) [16, 17]. Our results suggest that NETs ≤ 10 mm in diameter are better resected with ESMR-L than other endoscopic resection methods in terms of clinical efficacy, technical ease, and procedure time.

We included not only pathologically definite positive margin but also very small safe margin into incomplete resection. This is because there are not standard treatment guidelines for the histological findings that show a very small safe margin from the NET or near exposure to margin (‘possible’ remnant NET). Moreover, our results confirm with a previous prospective study by Sung et al. which reported a long-term good prognosis in possible remnant NET post endoscopic resection of a tumor less than 15 mm in diameter [19].

Of the 15 patients who were excluded because of the additional endoscopic resection that was carried out, eight patients showed a suspicious residual lesion. However, pathological results after additional resection showed no remaining tumor. Moreover, among the 31 patients with long-term follow-up, 24 cases had a clean scar in the resection site and seven cases had a scar with a visible lesion. However, all seven cases showing a suspicious residual NET were also confirmed to be negative after a follow-up biopsy. A previous study reported discrepancies between complete endoscopic resection and complete histological resection (100% vs. 75.3%) [19]. Stier et al. also reported that even in healthy scar, six out of 27 cases were confirmed to have residual NET after additional endoscopic resection [20]. These results suggest that the precision of predicting local recurrence by endoscopy can be low. Nevertheless, in our study, pathologically incomplete or very small margin NET showed a good prognosis. This suggests that positive resection margin is relatively safe in small rectal NET.

Our suggestions on the strategy when we receive a patient with incompletely resected small rectal NET, due to the limitations in consistency of the pathologist’s decision of margin involvement, we will first need to re-confirm the slide readings in our tertiary care institution, followed by endoscopic and EUS confirmation of the suspected residual lesion. If any of the clues show residual lesions, we will decide to re-perform endoscopic resection. To prove that this strategy is acceptable, a well-designed prospective study will be needed.

The exact suitable follow-up duration post incomplete resection has not yet been determined. In a 10-year retrospective study of long-term follow-up of 13 cases for possible remnant NET, only one patient showed local recurrence after 56 months during surveillance colonoscopy [19]. Moreover, there was a case report of liver metastasis 5 years after complete resection of an 8 mm sized G1 rectal NET [21]. In our study, neither local recurrence nor distant metastasis was observed over the 2-year follow-up period. However, considering previous reports and the slow growth rate of NET, a 2-year follow-up period cannot be suggested to be sufficient.

One of the limitations of this study is that it is a retrospective single-center study. Second, due to the strict inclusion criteria, only 31 patients were included which may not render the results generalizable to larger populations. Third, to collect data of as many patients as possible with longer follow-up periods, at least 2 years of follow-up was a maximal margin which seems to be a short period considering the slow growth rate of rectal NET. Furthermore, longer follow-up period of incomplete resection without additional treatment is warranted for the assessment of long-term prognosis. Fourth, we only used CT scans for staging and follow-up rather than MRI or Gallium-68 PET/CT, which may be more sensitive to detect small recurrences or small volume of metastatic disease [13]. Despite these limitations, to the best of our knowledge, our study is the first to demonstrate a good prognosis without performing additional resection in a pathological incompletely resected small rectal NET.

We can suggest that pathologically incompletely resected small G1 rectal NET can be safely observed without additional treatment in the first two years. However, the ideal follow-up intervals and duration should be further studied to confirm safety against late recurrence.

## Supplementary Information

**Additional file1: Table S1**. Clinical characteristics of excluded 21 patients who underwent additional resection.

## Data Availability

The datasets used and analyzed during the current study are not publicly available due to keeping privacy of patients but are available from the corresponding author on reasonable request.
