# Supplementary material for: Prognosis of incompletely resected small rectal neuroendocrine tumor using endoscope without additional treatment
Source: BMC Gastroenterol. 2022 Jun 9;22:293. doi: 10.1186/s12876-022-02365-z (PMC9185951; doi:10.1186/s12876-022-02365-z)
Supplement: Supplementary file 1 — Additional file1: Table S1. Clinical characteristics of excluded 21 patients who underwent additional resection. [file 12876_2022_2365_MOESM1_ESM.docx]

Supplement Table

Table 1. Clinical characteristics of excluded 21 patients who underwent additional resection

|  | Clinical clues |
| --- | --- |
| Additional endoscopic resection (n=15) | Residual lesion confirmed by EUS (2/15) |
|  | Suspected residual lesion by endoscopy (4/15) |
|  | None (9/15) |
| Surgical resection (n=6) | Grade 2 with Ki 67 index 3% (2/6)  None (4/6) |
